# CMDX©-based single source information system for simplified quality management and clinical research in prostate cancer

**DOI:** 10.1186/1472-6947-12-141

**Published:** 2012-12-03

**Authors:** Okyaz Eminaga, Mahmoud Abbas, Reemt Hinkelammert, Ulf Titze, Olaf Bettendorf, Elke Eltze, Enver Özgür, Axel Semjonow

**Affiliations:** 1 Department of Urology, University Hospital of Cologne, Kerpener Straße. 62, Cologne D-50937, Germany; 2 Institute for Pathology, University Hospital Hannover, Carl-Neuberg-Str. 1, Hannover D-30625, Germany; 3 Prostate Center, Department of Urology, University Hospital Muenster, Albert-Schweitzer-Str. 33, Muenster D-48149, Germany; 4 Prostate Center, Gerhard-Domagk Institute for Pathology, University Hospital Muenster, Domagkstr. 17, Muenster D-48149, Germany; 5 Institute of Pathology and Cytology, Technikerstrasse 14, Schüttorf D-48465, Germany; 6 Institute for Pathology, Saarbrücken-Rastpfuhl, Rheinstrasse 2, Saarbrücken D-66113, Germany

**Keywords:** Single source, Prostate cancer, cMDX, Heat map, Information system, Pathology report

## Abstract

**Background:**

Histopathological evaluation of prostatectomy specimens is crucial to decision-making and prediction of patient outcomes in prostate cancer (PCa). Topographical information regarding PCa extension and positive surgical margins (PSM) is essential for clinical routines, quality assessment, and research. However, local hospital information systems (HIS) often do not support the documentation of such information. Therefore, we investigated the feasibility of integrating a cMDX-based pathology report including topographical information into the clinical routine with the aims of obtaining data, performing analysis and generating heat maps in a timely manner, while avoiding data redundancy.

**Methods:**

We analyzed the workflow of the histopathological evaluation documentation process. We then developed a concept for a pathology report based on a cMDX data model facilitating the topographical documentation of PCa and PSM; the cMDX SSIS is implemented within the HIS of University Hospital Muenster. We then generated a heat map of PCa extension and PSM using the data. Data quality was assessed by measuring the data completeness of reports for all cases, as well as the source-to-database error. We also conducted a prospective study to compare our proposed method with recent retrospective and paper-based studies according to the time required for data analysis.

**Results:**

We identified 30 input fields that were applied to the cMDX-based data model and the electronic report was integrated into the clinical workflow. Between 2010 and 2011, a total of 259 reports were generated with 100% data completeness and a source-to-database error of 10.3 per 10,000 fields. These reports were directly reused for data analysis, and a heat map based on the data was generated. PCa was mostly localized in the peripheral zone of the prostate. The mean relative tumor volume was 16.6%. The most PSM were localized in the apical region of the prostate. In the retrospective study, 1623 paper-based reports were transferred to cMDX reports; this process took 15 ± 2 minutes per report. In a paper-based study, the analysis data preparation required 45 ± 5 minutes per report.

**Conclusions:**

cMDX SSIS can be integrated into the local HIS and provides clinical routine data and timely heat maps for quality assessment and research purposes.

## Background

**Figure 1:**
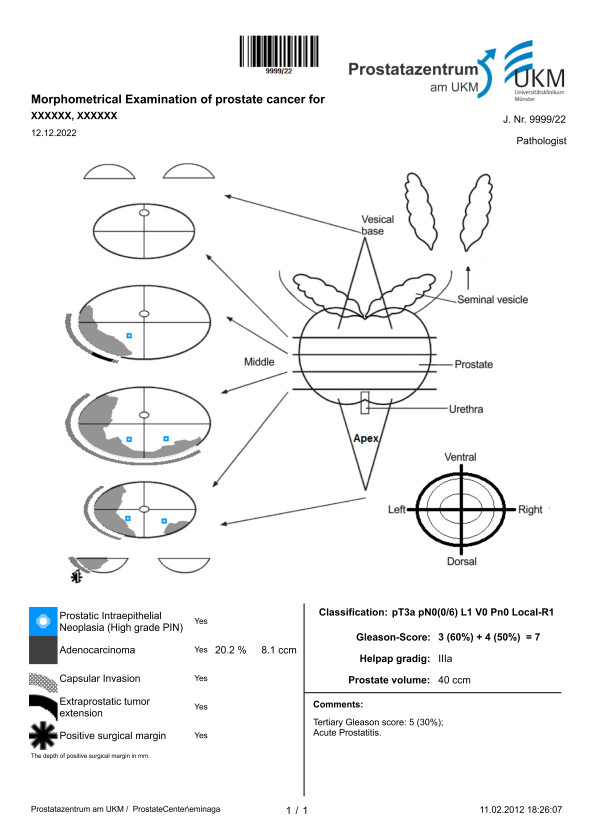
**Pathologic report of prostatectomy specimens.** An example of a morphometric mapping of a radical prostatectomy specimen showing an adenocarcinoma of the prostate in the left lobe with extracapsular tumor extensions and a positive surgical margin. In addition to the Gleason system, the tumors are graded according to Helpap. Here, prostate cancer takes up about 20.2% of the total area of the prostate, which corresponds to an estimated tumor volume of 8.1 cm^3^ in a prostate of 40 cm^3^. The computational estimation of tumor volume was performed as previously described by Eminaga et al [6].

A hospital information system (HIS) is a network-based multi-target computational system that facilitates information management to improve health care management. Physicians, researchers, patients, nurses, information technology engineers and administrators are all involved in the HIS. Thus, interdisciplinary interactions can be realized with helpful tools provided by the applied HIS. These interactions are essential for disease management, especially in cancer. In this case, we focus on prostate cancer (PCa), one of the leading cancers in men [1]. As a therapeutic approach, many patients diagnosed with PCa undergo total removal of the prostate gland (radical prostatectomy). The histopathological evaluation of prostatectomy specimens plays a crucial role in decision-making and predicting the patient’s outcome [2]. Consequently, diverse standardized sectioning and documentation protocols of radical prostatectomy specimens have already been introduced [2-4]. Here, we use a standardized protocol for pathologic reports according to Bettendorf et al. [4] (Figure 1). The report includes personal data and clinical data (i.e. tumor classification, grading and malignancy) and a diagrammatic representation of the histopathological findings in the prostate gland. It is a practical method for documenting tumor extension patterns and the status of the surgical margins in radical prostatectomy specimens. The surgical margin status is important for the assessment of treatment quality and disease management in the period after the primary surgical therapy [5]. Additionally, the anatomic diagram enables an approximate estimation of the absolute and relative tumor volume in the prostate [4,6]. The prostate tumor volume is an independent predictor for biochemical recurrence [7,8].

In the last decade, a stepwise transformation of paper-based patient records into electronic records has been accomplished in most clinics. Consequently, many researchers are now collecting data either from the HIS or from paper-based patient records for research purposes. Often these data are then stored in a separate data container (the Dual system). This form of data collection is time-consuming and can be affected by systematic errors. A study carried out by Ammenwerth et al. showed that physicians typically spend 26.6% of their working time in the clinic filling out documentation [9]. Furthermore, those physicians who are involved in clinical research spend more time with documentation than with their clinical duties and activities. Therefore, a well-defined approach is necessary to reduce data redundancy and the time required for documentation by reusing routine data for clinical research. A single source concept facilitates the reduction of data redundancy and the reuse of clinical data for research purposes [10].

The purpose of this article is to introduce the general concept and implementation process for a single source information system (SSIS) based on cMDX© (Clinical Map Document based on XML). cMDX is open-source, meets Open Packaging conventions and provides a data-acquisition model for graphical and textual clinical information. The graphical information is stored using a method based on scalable vector graphics. cMDX has already been applied to the reporting and analysis of PCa in prostatectomy specimens [6]. In this context, we will investigate its facility for reusing routine clinical data for research projects. Furthermore, we will evaluate the applied system information and compare it with the dual source information system. Our aim is to improve the information exchange between physicians from diverse medical disciplines and the analysis of routine health care data for research purposes and quality management.

## Methods

We began with the implementation of cMDX SSIS in the pathology institute and in the Department of Urology at University Hospital Muenster in Germany at the end of 2009. The implementation process was performed in three stages. First, we analyzed the clinical workflow for processing radical prostatectomy specimens and documentation of histopathological evaluation. Then we conducted unstructured interviews with physicians to learn their requirements and obtain their suggestions for improving the clinical workflow. Next, we designed the technical approach and assessed the technical requirements. Finally, a prospective study was conducted to evaluate the implemented information system.

### Workflow and report analysis

The diverse workflows and documentation procedures were analyzed using methods for business workflow analysis before and after implementation of cMDX SSIS.

### Pathological examination

Immediately after removal, the prostatectomy specimens were directly delivered to the pathology institute. The specimens were visually examined, inked, and fixed in buffered formalin (4%) for 24-48 hours. After fixation, the specimens were weighed with and without the seminal vesicles. According to what is routinely done in the clinic, the prostate weight in grams was equalized to its volume in cubic centimeters (cm^3^). A correction factor for tissue shrinkage after formalin fixation was ignored. Both seminal vesicles and the base of the seminal vesicles of every side were embedded separately. The urethral margin was cut as a 3-5 mm thick section parallel to the margin, which was serially sectioned at 2 mm intervals in parasagittal planes perpendicular to transverse cutting and parallel to a segment close to the urethra. The top margin was removed and sectioned in a similar way. The remaining prostate gland was serially sectioned at 5 mm intervals in transverse layers perpendicular to the anatomical course of the urethra. The sections of the prostate were divided into right and left halves and, depending on the size of the prostate, into a front and back part. The prostate was totally embedded. Blocks of each slice were sectioned at 5 μm and stained with hematoxylin and eosin to perform a microscopic examination. PCa was graded according to the Gleason grading system [11] as well as Helpap, and staged using the UICC TNM staging system [12].

### Documentation of histopathological evaluation

Bettendorf designed a schematic diagram to document the results of the histopathological evaluation of radical prostatectomy specimens [4]. The anatomical schema combines both seminal vesicles and a prostate gland divided into eight defined slices (Figure 1). Each slice expresses a fraction of the total prostate volume, which is defined as the slice factor. This percentage distribution was a consequence of internal pathologic observation of the prostate volume and the prostate dimensions. Symbols and icons are added to facilitate identification of the pathological findings, which enables documentation of the location of positive surgical margins (PSM) and PIN 3° (Prostatic Intraepithelial Neoplasia 3 Grade). Information such as tumor extension, areas of capsule infiltration as well extracapsular tumor extension was transformed from the representative slides to the diagrammatic schema of the prostate. Each pathology report is then assigned to a unique journal number with a text format “9999/YY” that consists of the year in short format and the serial number of the report generated in that year.

In cMDX SSIS, the creation of electronic reports and the computational estimation of tumor volume are performed using the cMDX Editor software, as previously described by Eminaga et al. [6]. Prior to that, the histopathological evaluation was documented in paper-form, and the tumor volume (TV) was qualitatively estimated by visual judgment. The Interclass Correlation Coefficient (ICC) was calculated for assessing the intra- and inter-observer reliability.

### Clinical decision making

As an example of the typical clinical decision making workflow, physicians from different medical disciplines (urology, radiology and pathology) come together at the weekly interdisciplinary tumor conference, where prostate cancer cases are presented. During the tumor conference, participants review the pathology report and make their decisions about aftercare for convalescent patients depending on the clinical data. The aftercare may include regular examination at defined periods, radiation therapy and/or hormone therapy. The names and specialties of the participants, and intended procedures are documented in the conference protocol. The procedures are documented and stored in the associated patient records. After that, the urologist visits with the affected patients and conducts a personal conversation with them about further procedures with respect to personal privacy. Finally, the aftercare is organized according to treatment conditions and the clinical decision.

### Technical approach

**Figure 2:**
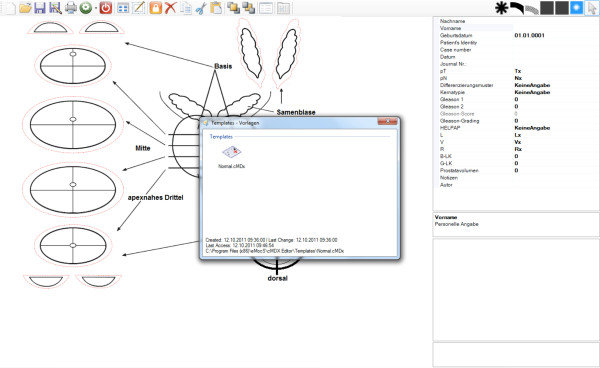
**Graphical User Interface of the cMDX Editor.** The main window of the “cMDX Editor” software.

**Figure 3:**
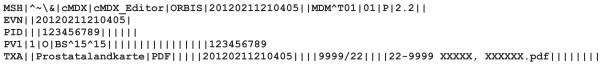
**Example of a HL7 MDM message.** “cMDX Editor” generates this message to transfer the pathology report into the corresponding electronic health record.

We used the infrastructure of the existing information system to establish cMDX SSIS. A virtual machine was built using the VMware Server© software provided by the Data Center of University Muenster. The virtual server includes the Microsoft© Windows® XP Professional Edition operating system and cMDX Editor (Figure 2). This virtual server is accessible within the internal network through the remote desktop. In addition, electronic reports generated with cMDX Editor were stored on a local network hard drive. The cMDX Editor is a c# based program and offers functions to manage cMDX Documents. The software encrypts sensitive data with symmetrical 256-bit rijndael cryptography [13] to avoid data misuse by unauthorized users. The generated reports are stored in the PDF and cMDX file formats. The file name consists of the journal number of the corresponding report and the patient name. In Addition, cMDX Editor sends a HL7 MDM message (Health Level 7 Medical Document Management) to the current hospital information system to transfer the pathology report saved as a PDF file into the corresponding electronic health record (HER) in Orbis™ (Figure 3). For this purpose, the case number or the patient identification number generated by Orbis are obligatory.

### Data evaluation

In our department, we performed a prospective study between Jan. 2010 and Dec. 2011 to evaluate the suitability of cMDX SSIS for clinical routine use and research. The study was made in compliance with the World Medical Association Declaration of Helsinki on Ethical Principles for Medical Research Involving Human Subjects [14]. Patients gave their informed consent before participating in this study. In addition, local German regulations allow the use of clinical data collected in a university hospital setting for research purposes. Access to the HIS data was only authorized by the treating physicians and the director of the clinic and all extracted HIS data were anonymized.

We evaluated the time and steps necessary for complete documentation and for performing analysis of the cMDX files in the clinical study. We analyzed the source-to-database error according to Nahm et al. [15] and the completeness according to Chan et al. [16] to describe data quality. In addition, we compared our approach with the method applied in a study by Koepke et al. [17] to generate a cumulative heat map of the distribution of PCa, as well as with our recent retrospective study based on cMDX [6]. The data collection of both studies was performed according to the dual source system.

## Results

The documentation of the pathologic reports was analyzed and it was determined that types of information exist: textual and graphical. There are 30 inputs fields that can be answered via the drawing area, the combination box with three to four values or with the text box. The graphical information is stored as a vector graphic. We developed the cMDX Editor and implemented it in the clinic over 6 months to achieve a full electronic workflow. The numbers on usage in this paper are mainly based on the data received between Jan. 2010 and Dec. 2011.

### Paper-based and cMDX-based documentation workflow

The pathology reporting workflow before implementation in the HIS began after finishing the histopathological examination. The pathologist enters the clinical information, draws the spread pattern of PCa and marks the location of PSM and PIN3 on the mapping designed by Bettendorf. This pathologic information is then used for disease management. When completed, the pathologist makes two copies of the report for archiving and for the tumor conference. The volume estimation of PCa is made by visual estimation and the malignancy grade (HELPAP) is calculated manually. The report is then filed in the patient’s medical record. The textual data are manually entered into a separate research database for scientific purposes. The PCa volume estimation was not regularly performed in the clinic before the implementation of cMDX SSIS. Therefore, in most cases the PCa volume had to be estimated for research projects. The Interclass Correlation Coefficients (ICC) for the estimation of tumor volume for one typical single pathologist and for different pathologists (EE, OB, MA) were 0.988 (95% CI: 0.982-0.993) and 0.999 (95% CI: 0.998-0.999), respectively.

**Figure 4:**
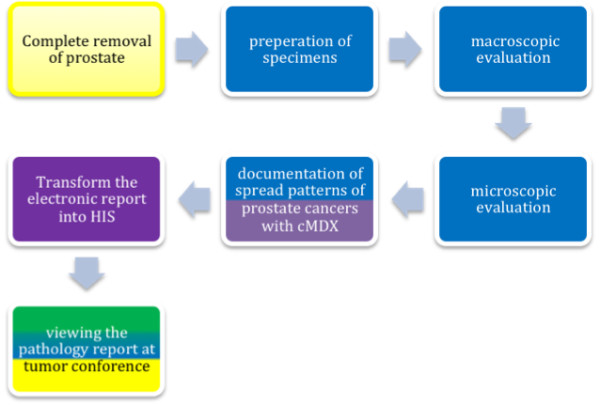
**Workflow of cMDX documentation after the implementation of cMDX SSIS in HIS.** After complete removal of the prostate, the specimen is prepared for histopathological examination. After that, the pathological information and the tumor extension are documented using the cMDX Editor. The cMDX Editor transformed the PDF file to the corresponding electronic health record. This cMDX report is also presented during the tumor conference. The background colors symbolize the involved medical disciplines and software: Yellow represents urology, blue for pathology, green for radiology, and lilac for cMDX Editor.

In this project, cMDX-templates of the pathology report were implemented in the local HIS and the medical staff received on-the-job-training. The training duration was 15 minutes and comprised all aspects related to the accessing and application of the cMDX templates available in the HIS. Two pathologists participated in this training. In the new workflow after the histopathological examination, the pathologist calls the program “cMDX Editor” and selects the needed template. The pathologist then documents the results in the cMDX report. The documentation of the pathology report took approximately 10 ± 2 minutes. In addition, the software performs the computational volume estimation of PCa. The report is stored in a defined network drive after the user confirms the Save command. During the meeting of the tumor conference, the reports are projected on the display wall and are viewed by participants. If the physician enters the related patient’s case ID, the report is stored in the patient’s electronic health record (EHR). Furthermore, the software can print the report automatically, if the user activates this function in the “option setting”. In case the pathologist has to complete or modify the report, he/she can call up the desired report from the network drive. All reports are accessible for authorized users within the HIS and the user can search for the required report by the journal number. Figure 4 demonstrates the workflow of cMDX documentation after the implementation of cMDX SSIS in the HIS has been completed.

### Data quality

**Figure 5:**
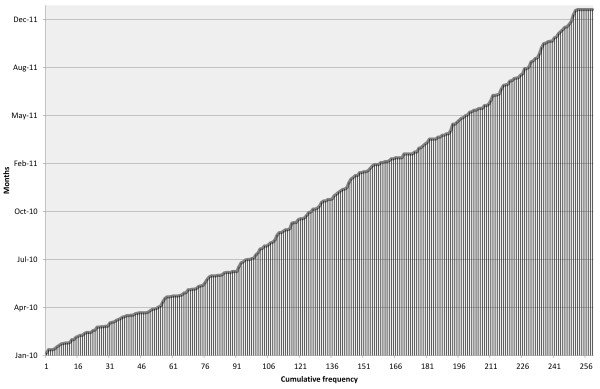
**Cumulative frequency of cMDX-based pathology reports since Jan. 2010.** A noticeable increase can be seen in the number of cMDX-based pathology reports.

We focused on the mandatory items (e.g. TNM classification system, Gleason score, and tumor volume) for pathology reporting to assist the report completeness rate. During a two-year period (Jan. 1, 2010 until Dec. 31, 2011) we matched the number of patients who underwent radical prostatectomy with the number of completed pathology reports. In addition, at one-month intervals we determined the number of pathology reports generated in the defined period to assess the user acceptance of cMDX SSIS. Two hundred fifty-nine patients with prostate cancer were treated with radical prostatectomy. At same time, 259 cMDX-based pathology reports were generated in the study period. The number of cMDX reports was increased monthly (Figure 5). All pathology reports included all mandatory items. Therefore, we achieved a report completeness rate of 100% by using cMDX SSIS. Before applying the automatic file naming to the cMDX reports, a total of 4320 data fields were audited; six reports had an error in the file name and the journal number, which were corrected without any problem; this represents a source-to-database error rate of 13.9 errors per 10,000 fields. After implementing the automatic file naming in 2011, we identified only 2 errors in 3450 fields, yielding an estimated error rate of 5.8 errors per 10,000 fields. The difference between both error rates is significant (p < 0.000).

We transferred 1623 pathology reports generated between 1999 and 2009 into cMDX format; 10 pathology reports were not readable. The prostate volume of each patient was not available in the pathology report and had to be completed from another data sheet.

### Simplified use in clinical research

**Figure 6:**
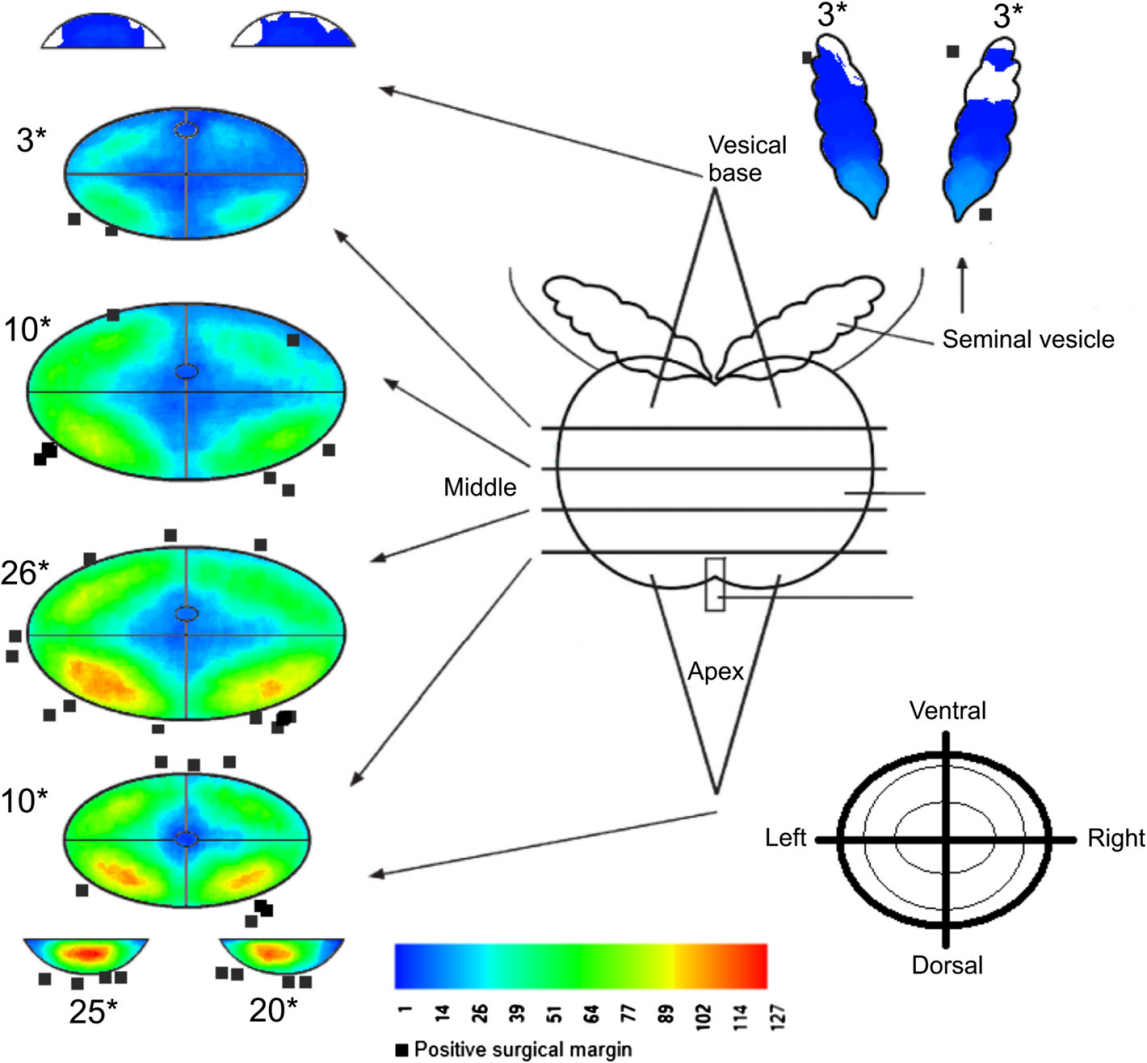
**Heat map diagram representing the patterns of spread of prostate cancer and positive surgical margins.** The cumulative diagram of spread patterns of prostate cancer in 259 prostatectomy specimens. The color gradient represents the frequency rate of PCa foci in the various locations (blue: low frequency; red: high frequency). * denotes the frequency rate of positive surgical margins in each slice.

A total of 259 reports were enrolled in our study. The cMDX reports were evaluated by an analysis tool called “cMDX Analyzer”. Acquired data were then exported as CSV files (comma-separated values) into the statistical program IBM SPSS Statistic 19 (SPSS Inc., Chicago, IL, USA) to perform descriptive statistics to characterize the patient population (Table 1). The mean relative tumor volume was 16.6% (range: 0-63.1%). The analysis tool generates a color diagram representing the spread patterns of prostate cancers (Figure 6). The accuracy and precision of these spatial localizations was evaluated on 10 prostatectomy specimens. No deviation in the location of PCa foci was found in the heat map in comparison to the original reports. In most cases, PCa affects the posterior lateral region of prostate. The positive surgical margins are mostly localized in the apical region of the prostate. As a result, researchers can reuse routine medical data stored in cMDX SSIS for study. The analysis of all 259 reports took an average of 26 ± 3 sec on a notebook computer with the following hardware specifications: Intel© i5 Core, 4 GB 1333 MHz DDR3 RAM, 128 GB SSD, Intel© HD Graphics 3000 with 384 MB DDR3 SDRAM.

**Table 1:** Cohort characteristics

	**Frequency (%)**
Patients	259 (100)
Pathologic tumor stage	
pT1a	7 (2.7)
pT2a	26 (10.0)
pT2b	5 (1.9)
pT2c	132 (51.0)
pT3a	52 (20.1)
pT3b	36 (13.9)
ypT4	1 (0.4)
Lymph node status	
pNx	1 (0.4)
pN0	237 (91.5)
pN1	21 (8.1)
Surgical margin status	
Rx	2 (0.8)
R0	197 (76.1)
R1	60 (23.2)
Lymph vessel involvement	
Lx	1 (0.4)
L0	188 (72.6)
L1	70 (27.0)
Vein involvement	
Vx	1 (0.4)
V0	235 (90.7)
V1	23 (8.9)
Gleason Score	
5	5 (1.9)
6	53 (20.5)
7	151 (58.3)
8	5 (1.9)
9	43 (16.6)
Cannot be estimated	2 (0.8)
HELPAP	
Ib	4 (1.5)
IIa	78 (30.1)
IIb	96 (37.1)
IIIa	26 (10.0)
IIIb	53 (20.5)
Cannot be estimated	2 (0.8)

Additionally, during the study we estimated the time elapsed from generation of the pathology report of prostatectomy specimens to receiving the report by the urologists and then to data analysis. The cMDX reports are available and accessible immediately after the documentation is completed. By contrast, the paper-based reports were mostly received and previewed by urologists for the first time during the weekly tumor conference.

The data acquired in cMDX SSIS can be analyzed and reused in a clinical study without any major preparation. Recently, the pathology reports were stored in the corresponding patient records only in paper form. In our last study, 255 of 1623 paper-based pathology reports generated between Jan. 1999 and Sep. 2009 were transformed into electronic form. All reports were scanned and saved as images (i.e. JPEG or BMP); the images were processed using freeware photo editing software called Paint.Net (dotPDN LLC, USA) [17]; here, the PCa foci had been distinguished from other shapes and were highlighted. Then, we converted these images into cMDX document format using cMDX Editor (Additional files 1 and 2). The whole process took 15 ± 2 minutes per report on average.

Before developing cMDX, the pathology reports were copied with a magnification factor of 141% on transparent follies. These follies were then holed on the computer screen, where a commercial spreadsheet program was also opened. The spreadsheet contains the defined input areas representing the anatomical schema of the pathology report. The PCa foci were transferred manually into the corresponding areas on the display screen as in the original. This process tool approximately 45 ± 5 minutes per report in average.

## Discussion

With cMDX SSIS, it is feasible to generate heat maps of the spread patterns of PCa from routine data. The analysis tool enables the analysis of graphical data to obtain a resulting image file with the heat map and CSV files containing clinical data and analysis results. The data analysis is performed in compliance with data privacy regulations. The applied tools are user-friendly and well-accepted by physicians. Therefore, cMDX SSIS is a practical approach to obtain timely heat maps and results from the current data without any major preparation. Previously, the physicians had to fit data from routine documentation to perform analysis. This procedure is time-consuming and can cause systematic errors during data fitting. The goal of improving the primary information system is well known, but the implementation of single source systems is not common [10]. We conducted a PubMed search using the key words “heat map and prostate cancer”, “heat map and documentation” and “map and prostate cancer”. Here, we did not find any similar approaches to the implementation of this form of documentation in the HIS that can provide timely heat maps.

The anatomical representation of the prostatic gland enables the collection and analysis of the spread pattern of PCa as well the status of the surgical margins. Such information is necessary to optimize the biopsy strategy, so that the detection rate of PCa can be increased [18-20]. In addition, the status of the surgical margins plays an essential role in treatment and quality management assessments, which is one of the important conditions for certification of prostate centers [5]. The tumor volume is presumably an important prognostic indicator for predicting prostate cancer recurrence following surgery and therefore must be mentioned in the pathologic reports [8]. The pathology report can be denoted as a supportive tool for decision-making in cancer management and therefore must be clearly structured and informative for physicians. With cMDX SSIS, we have achieved the creation of pathology reports within a short turnaround time.

The measured data quality, especially the completeness of the documented items per form, is high. The completeness of the electronic forms in the HIS (current documentation) was found to be 100%. We presume that clinical routine data are documented regularly in each patient. By contrast, the clinical data not related to the clinical routine are commonly not documented; however it is this kind of data that is regularly acquired for research purposes. Furthermore, the retrospective study seems to have lower data completeness than the prospective study because in a retrospective study, patients with missing or incomplete data may be present. According to Chan et al., the data completeness varied considerably across studies and that even in the same organization, the amount of missing data was variable [16]. Nahm et al. concluded that medical record abstraction is the most significant source of errors and should be controlled and managed during the course of clinical trials; the acceptance criteria for analytical variables were 10 errors per 10,000 fields and zero errors per 10,000 fields [15]. The source-to-database error rate in the documentation process is acceptably low (on average, 10.3 errors per 10,000 fields). We assume that the regular evaluation of pathology reports during the tumor conference is the main reason for the low error rate. Since applying the automatic file naming of cMDX reports, the source-to-database error rate was decreased significantly. Therefore, the automation of standardized well-defined procedures may reduce the error rate in the documentation process. Nevertheless, further investigations are needed to validate this statement.

The transformation of paper-based documentation to electronic form is time consuming and mostly associated with data incompleteness. Furthermore, additional data sources, if available, are necessary to achieve data completeness.

cMDX SSIS was implemented in a commercial HIS and is therefore applicable to all other customers using the same HIS. In addition, cMDX supports standard document formats such as HL7 and CDA (clinical document architecture) and can store pictures and images [6]. The pathological information in prostatectomy specimens is now available in the HIS for many patients. Attributes such as tumor staging, Gleason score, and tumor extension are documented in a structured way, which can be used as inclusion or exclusion criteria for patient recruitment in clinical trials. The supplementary cMDX Editor tool is not currently available for public download, but the interested reader can contact the corresponding author to obtain a copy of the tool for use.

### Limitations

This article focused primarily on pathology reports of prostatectomy specimens. The statement of transferability of cMDX SSIS to other clinical fields is therefore limited. The technical approach described here may be accompanied by technical failures, which requires appropriate handling to keep the system working without interruption. Any technical interruption could result in data incompleteness and disrupted workflows in clinical routines. Thus the system requires continual monitoring and periodic maintenance.

## Conclusions

cMDX SSIS facilitates the electronic documentation of pathology reports. Implementation of this system into the local HIS is feasible; it enables the reuse of the pathological information for quality management and research purposes as well for generating timely heat maps from routine clinical data.

## Competing interests

The authors declare that they have no competing interests.

## Authors’ contributions

OE implemented the cMDX reports, developed supplementary tools, analyzed data quality and wrote the manuscript. AS, MA, EE, OB, RH and UT provided clinical data information. AS and EO critically revised the manuscript. All authors read and approved the final manuscript.

## Pre-publication history

The pre-publication history for this paper can be accessed here:

http://www.biomedcentral.com/1472-6947/12/141/prepub

## Supplementary Material

Additional file 1 **An example of a pathology report in paper form.** This file can be viewed with Adobe Acrobat Reader. Click here for file

Additional file 2 **The electronic version of the pathology report.** The paper version of this report is given in Additional file 1. This file can be viewed with Adobe Acrobat Reader. Click here for file
