# Supplementary material for: CMDX©-based single source information system for simplified quality management and clinical research in prostate cancer
Source: BMC Med Inform Decis Mak. 2012 Dec 3;12:141. doi: 10.1186/1472-6947-12-141 (PMC3519791; doi:10.1186/1472-6947-12-141)
Supplement: Additional file 1 — An example of a pathology report in paper form. This file can be viewed with Adobe Acrobat Reader. [file 1472-6947-12-141-S1.pdf]

Kopie als Anlage
zum Entlassungsbericht

Pathologischer Befund Prostatakarzinom

UKM Universitätsklinikum Münster

Name: Geb. Dat.

J. Nr.

Prostatazentrum am UKM

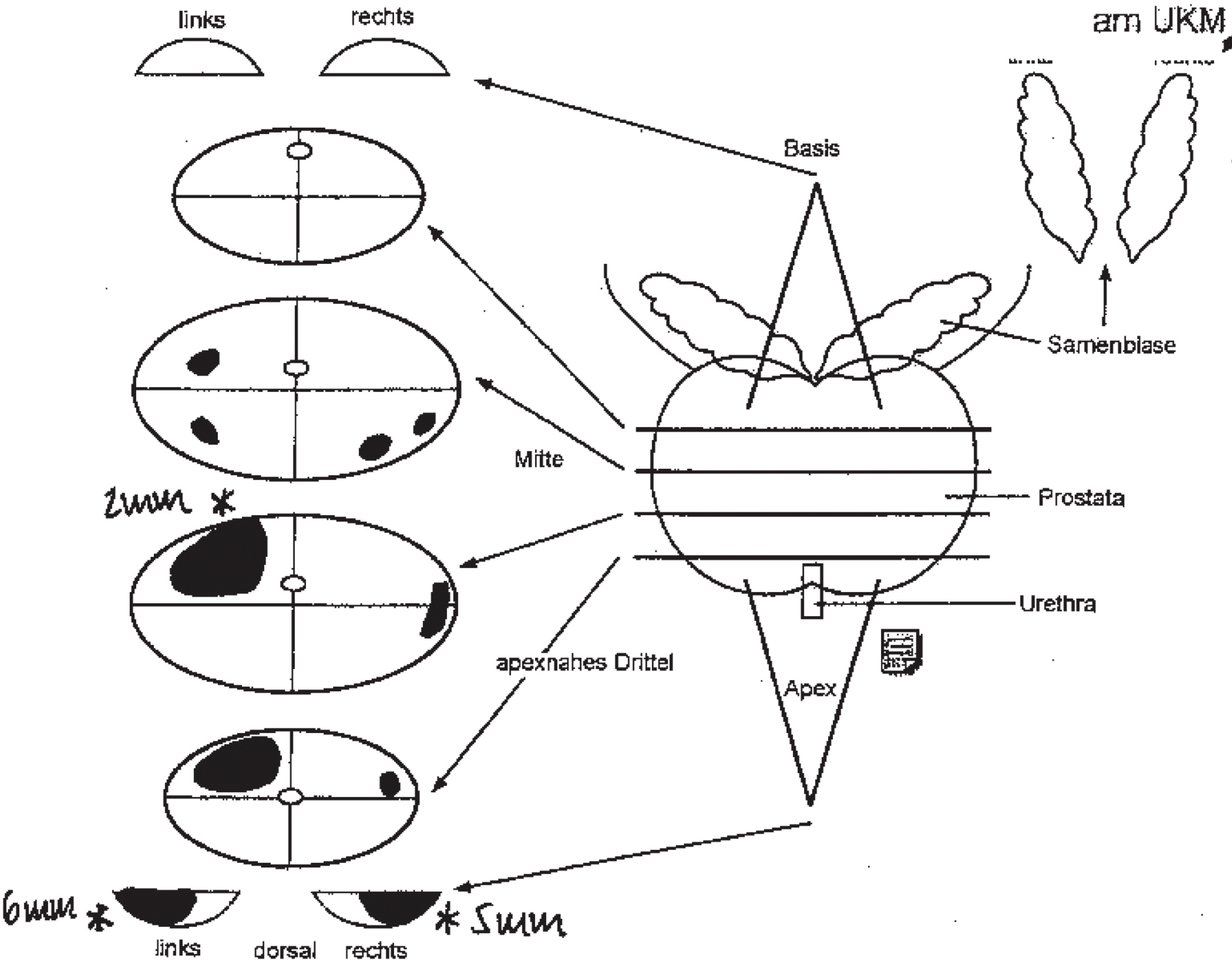

P	Prostatische Intraepitheliale Neoplasie (PIN) 3°	*Differenzierungsmuster*		*Kernatypie*	
	Adenokarzinom	☐ hoch differenziert	0	☐ gering	0
	Kapselinvasion	☐ wenig differenziert	1	☐ mäßig	1
	Extraprostatische Ausbreitung	☐ kribriform	2	☒ stark	2
*	positiver chirurg. Schnittrand	☒ solide	3		
		Ia = 0 Ib = 1 IIa = 2 IIb = 3 IIIa = 4 IIIb = 5			

pT: 2c	pN: 0 (0/5)	Gleason Score: 5+3 = 8 (2,4)	Malignitäts Grad (Helpap): III b
