# Supplementary material for: CMDX©-based single source information system for simplified quality management and clinical research in prostate cancer
Source: BMC Med Inform Decis Mak. 2012 Dec 3;12:141. doi: 10.1186/1472-6947-12-141 (PMC3519791; doi:10.1186/1472-6947-12-141)
Supplement: Additional file 2 — The electronic version of the pathology report. The paper version of this report is given in Additional file 1. This file can be viewed with Adobe Acrobat Reader. [file 1472-6947-12-141-S2.pdf]

Pathologischer Befund Prostatakarzinom für

,
01.01.0001

J. Nr.

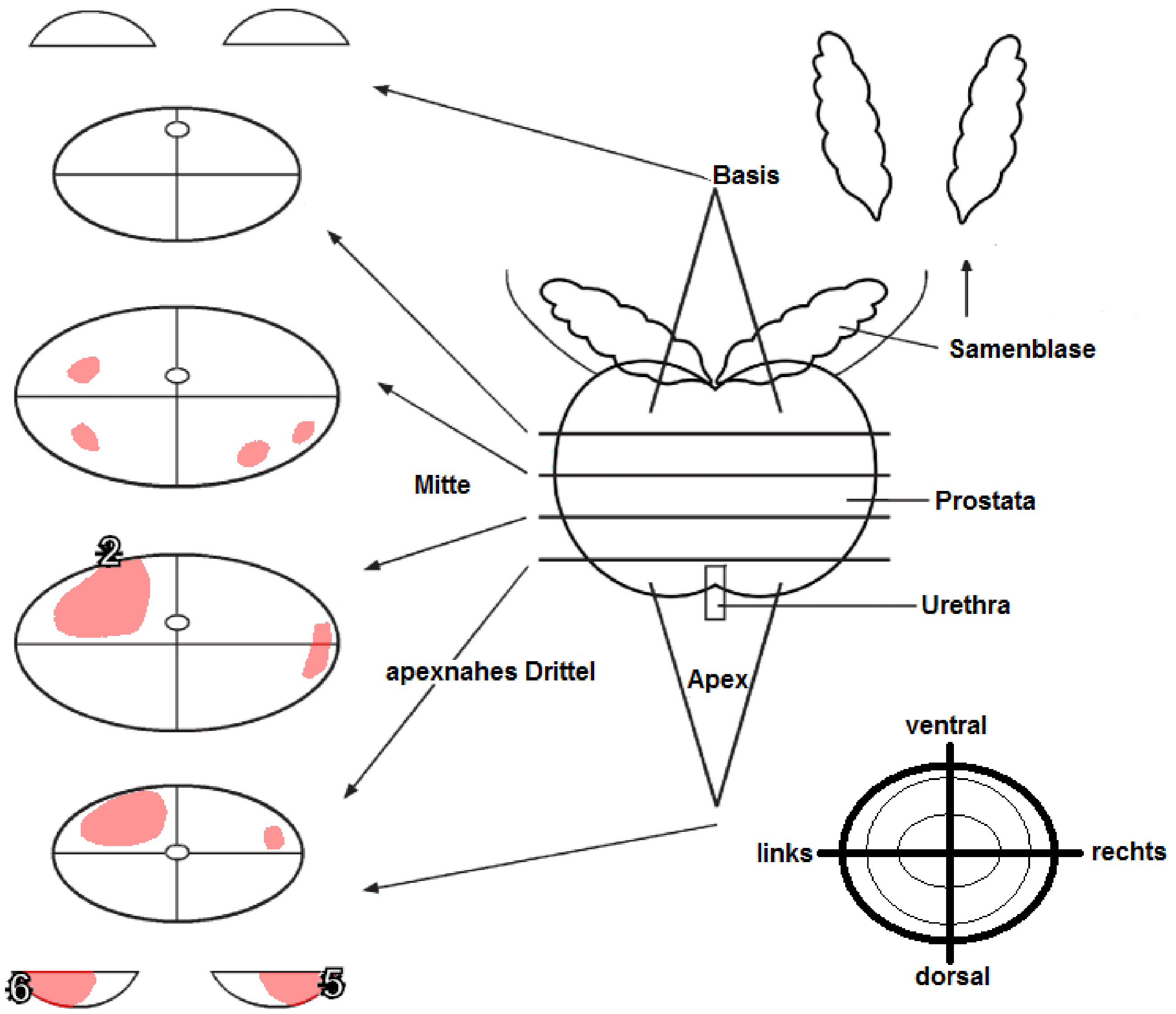

	Prostatische Intraepitheliale Neoplasie	Nn	
	Adenokarzinom	Ja	11,3 %
	Kapselinvasion	Nn	
	Extraprostatische Ausbereitung	Nn	
	positiver chirurg. Schnittrand	Ja	

Die Länge von positiven Schnitträndern ist in mm angegeben.

Klassifikation: ***pT2c pN0(0/5) Pn0 L0 V0 Lokal-R1***

Gleason-Score: 5 + 3 = 8

Malignitätsgrad: IIIb

Prostatavolumen: 0 ccm

Kommentar:
